# Mixed-type intraductal papillary mucinous neoplasm: Tailored surgical planning - case report

**DOI:** 10.1016/j.ijscr.2020.09.104

**Published:** 2020-09-24

**Authors:** Thiago Bassaneze, Alberto Youssef Laham, Luiz Guilherme Lisboa Gomes, Bruna Queiroz Coelho, Carlos Augusto Real Martinez

**Affiliations:** a Digestive Surgery Department, Hospital Brazil - Rede D’Or, Santo André, SP, Brazil; b Digestive Surgery Department, Faculty of Medicine of ABC Foundation, Santo André, SP, Brazil; c Post Graduate Program in Health of Sciences, São Francisco University Medical School (USF), Bragança Paulista-SP, Brazil; d Department of Surgery, University of Campinas, Campinas, SP, Brazil

**Keywords:** Case report, Pancreatic neoplasm, Pancreatic intraductal neoplasm, Pancreaticoduodenectomy, Immunohistochemistry, Mucin-2

## Abstract

**Introduction:**

With the greater availability of imaging exams, the diagnosis of intraductal papillary mucinous neoplasms (IPMNs) has increased recently. However, there are still questions about adequate management approaches for this disease, especially regarding the best therapeutic strategy. The objective is to describe the case of a patient with mixed-type (MT) IPMN successfully treated by a tailored surgical plan that adopted duodenopancreatectomy and imaging to monitor the remaining lesions of the tail and body of the pancreas.

**Presentation of case:**

A 65-year-old asymptomatic man underwent ultrasonography of the abdomen and was diagnosed with a cystic tumor, measuring 3.0 × 2.5 cm, located on the head of the pancreas. Magnetic resonance cholangiopancreatography (MRCP) showed dilation of the main pancreatic duct and multiple cystic lesions scattered throughout the entire parenchyma. The patient underwent duodenopancreatectomy; postoperatively, he did not have complications and was discharged on the 6th postoperative day. The histopathological panel confirmed the presence of MT-IPMN of the intestinal pattern. The patient is currently well four years after surgery and is undergoing semiannual MRCP examinations to follow up the remaining lesions.

**Discussion:**

MT-IPMNs represent 28–41% of all IPMNs. Among all subtypes, MT-IPMNs are the most challenging in terms of choosing the ideal therapeutic strategy. These lesions are the most difficult to treat because they can be multifocal and compromise different locations of the pancreatic parenchyma.

**Conclusion:**

MRI findings, Ca19.9 serum level and negative family history of pancreatic neoplasia were indications for the surgical choice for the MT- IPMN presented in this case.

## Highlights

• The diagnosis of IPMN has increased in recent years.
• Correctly identifying Mixed-Type IPMNs is related to the potential of these lesions for malignant transformation.
• Decision making and meticulous follow-up of the remaining pancreatic parenchyma should be considered.

## 1 Introduction

Intraductal Papillary Mucinous Neoplasms (IPMNs) are cystic lesions with mucin-producing cells that develop from the pancreatic ducts. These cystic neoplasms are more common after the fifth decade of life, affecting both sexes in similar proportions [1,2].

Decision making in the face of mixed-type IPMN (MT-IPMN), when the tumor compromises both main pancreatic duct and its secondary branches, has been a topic of debate in the last decade due to the challenges involved in selecting conservative or surgical management. In view of the difficulty in deciding between resection - with a possible increased risk of postoperative complications and decrease in organ function - and expectant treatment, the best strategy remains controversial [3,4].

This paper aims to report a case of MT-IPMN successfully treated by a tailored surgical plan that adopted duodenopancreatectomy and observation of the residual pancreatic parenchyma, with clinical considerations and a brief literature review. This work has been reported in line with the SCARE criteria [11].

## 2 Presentation of case

**Fig. 1:**
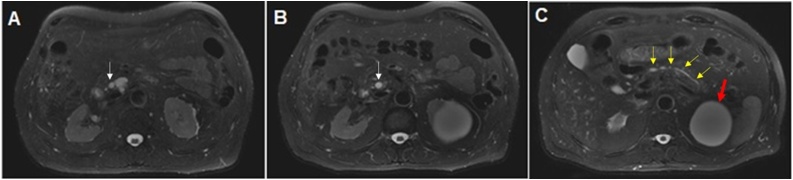
Axial fat-suppressed T2-weighted MRI sequences. **A:** Presence of a BD-IPMN located in the head of the pancreas measuring 22 × 17 mm (white arrow) **B:** MD-IPMN measuring 10.4 mm extending from the head to the neck of the gland (white arrow). **C:** Normal main pancreatic duct in the pancreatic body and tail (yellow arrows) and large left renal cyst classified as Bosniak 1 (red arrow).gr1

**Fig. 2:**
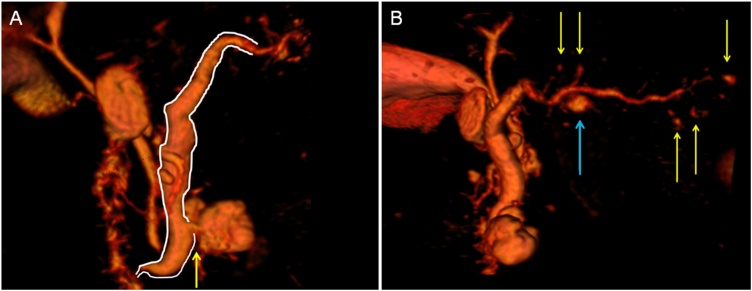
3D MRI **A:** MD-IPMN in the area marked by the white line and BD-IPMN in the pancreatic head (yellow arrow) **B:** Several BD-IPMNs located across the entire gland (yellow arrows), with a maximum diameter of 7.5 mm in the proximal pancreatic body (blue arrow). 3D Slicer Program - version 4.5 - USA [5].gr2

A 65-year-old male had no family history of pancreatic cancer in his family. He was treated in the Hospital Brazil - Rede D’Or, Santo André-SP, Brazil. He sought specialized care after undergoing a routine abdominal ultrasound scan that identified the presence of a cystic lesion, measuring 3.0 × 2.0 cm, located at the head of the pancreas with homogeneous signal and no septa. He denied experiencing abdominal pain, diarrhea, jaundice, choluria, and acholia as well as previous episodes of pancreatitis or weight loss. Physical examination revealed no abdominal distension or masses. The tumor markers showed a CEA value of 2.0 ng/mL and a Ca 19.9 value of 7.0 IU/mL. The patient’s CT scan of the chest and abdomen revealed no metastasis. Magnetic resonance cholangiopancreatography (MRCP) showed that the main pancreatic duct had cephalic ectasia, with a maximum dilation of 10.4 mm, and revealed the presence of a cyst, measuring 22 × 17 mm, located in the head of the pancreas that communicated with the main pancreatic duct (Fig. 1, Fig. 2).

After a multidisciplinary meeting, for the diagnosis hypothesis of MT-IPMN mainly involving the head of the pancreas, in a patient who did not have a family history of pancreatic carcinoma, surgical treatment was considered to be the best option. During surgical planning, it was decided that the main-duct IPMN (MD-IPMN), located in the head of the pancreas, would be removed by duodenopancreatectomy, and the branch-duct IPMN (BD-IPMN) would be kept in the nonresected portion of the organ and followed up with regular imaging exams.

**Fig. 3:**
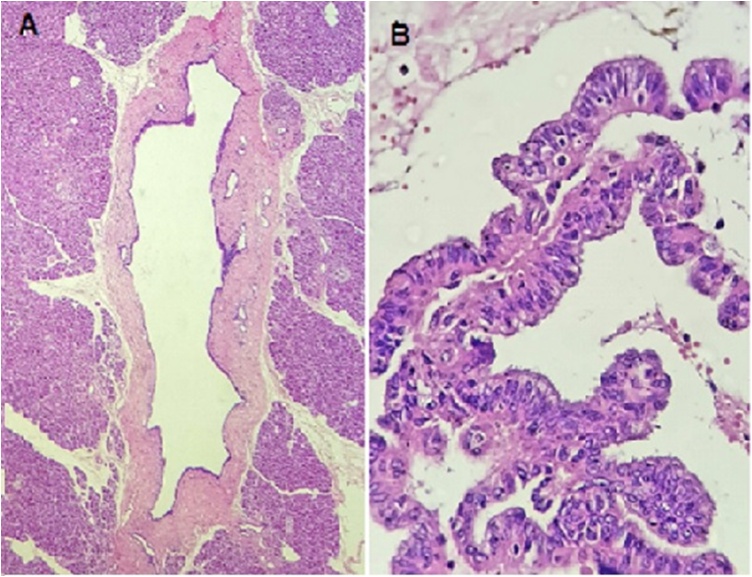
**A**: Transverse margin of the frozen sample of the main pancreatic duct, at the height of the gland neck, without evidence of neoplastic involvement (HE 100 ×). **B:** MD-IPMN with high-grade dysplasia; it is possible to identify cell pleomorphism with intense architectural disarray and budding into the main pancreatic duct lumen, without invasion or rupture of the lamina propria (HE 200 ×).gr3

During surgery, the gland was soft, and the cyst located in the pancreatic head was easily identified. There were no signs of regional lymph node involvement or peritoneal dissemination. A histopathological study of a frozen sample was performed to analyze the surgical resection margin at the height of the pancreatic neck. The examination showed that the main pancreatic duct was free from disease involvement (Fig. 3A). The surgical procedure was uneventful, performed by laparotomy, with minimal blood loss and lasted 6 h. The patient did not need blood transfusions during surgery or postoperatively. After resection of the organ, the intestinal transit was reconstructed using a single intestinal loop. We chose to perform pancreaticojejunal anastomosis using the duct-mucosa technique with 5.0 monofilament suture, biliodigestive anastomosis with termino-lateral hepaticojejunostomy and gastroenteroanastomosis in two planes. The patient recovered well in the postoperative period and was discharged after 6 days of hospitalization.

The histopathological study of the surgical specimen showed the presence of MD-IPMN located in the head of the pancreas with areas of high-grade dysplasia (Fig. 3B) and several other BD-IPMNs with low-grade dysplasia, the largest being in pancreatic head and measuring up to 22 mm. The immunohistochemical panel showed the presence of goblet cells with intense cytoplasmic expression for MUC2 and MUC5AC proteins in the areas with high-grade dysplasia and the absence of staining for MUC1, MUC6 and SMAD4 proteins (intestinal-IPMN subtype).

**Fig. 4:**
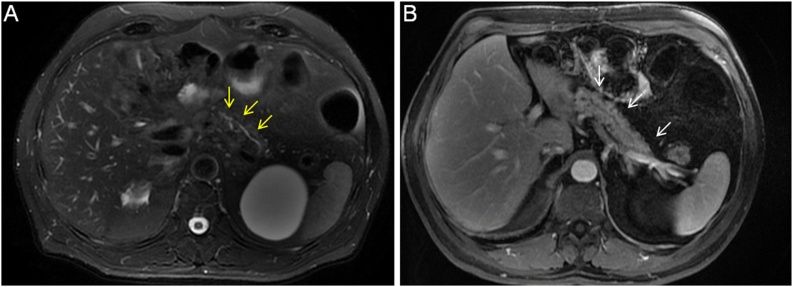
**A:** Axial fat-suppressed T2-weighted MRI sequence four years after surgery: main pancreatic duct with a normal caliber and without progression of the disease or new pancreatic cystic lesions (yellow arrows). **B:** Axial fat-suppressed T1-weighted MRI sequence: pancreatic remnant (white arrows).gr4

Currently, the patient is undergoing outpatient follow-up and undergoes MRCP every six months; this is the fourth postoperative year, and there are no signs of disease recurrence in the remaining pancreatic segment. The BD-IPMNs located in the nonresected body and tail remain subcentimeter in size without any increases in size (Fig. 4).

## 3 Discussion

### 3.1 Epidemiology

The exact incidence of the disease is unknown, but it is estimated that 2.6% of routine radiological studies of the abdomen detect pancreatic cysts incidentally, although it is unclear what proportion of these represents IPMNs [13]. IPMNs were first described in 1982 by Kazuhiko Ohhashi of the Japanese Foundation of Cancer Research as a “mucin-producing tumor of the pancreas” [17]. However, until the end of the 20th century, IPMNs were considered an uncommon entity but were among the most discovered and surgically resected pancreatic cystic lesions [1]. These lesions are usually located in the head of the pancreas, but multifocal involvement is often described [4]. These cystic neoplasms are more common after the fifth decade of life, affecting both sexes in similar proportions, and can cause dilatation of the pancreatic ducts [2].

### 3.2 Histology

Invasive carcinoma can develop both at the site of the IPMNs and at a different one, which is the main reason why most studies propose a detailed investigation of the entire pancreatic gland, including for remnant pancreas disease after an eventual partial pancreatectomy [[7], [8], [9]]. These findings associated with molecular studies of IPMNs support the concept of a “field defect” and show evidence that multifocal IPMNs are clonally distinct from each other [7,12].

IPMNs can present different degrees of cellular atypia and present a possibility of malignant transformation into intraductal carcinoma. Undoubtedly, all IPMNs harbor a risk of malignant transformation, but each type of IPMNs carries a different degree of malignant potential, and several clinicopathologic characteristics, including macroscopic type (differential involvement of the pancreatic duct system), seem to predict the risk of malignant transformation [1,2]. IPMNs with high-grade dysplasia have the possibility of progressing to invasive ductal adenocarcinoma. IPMNs with low-grade dysplasia have a lower chance of malignant transformation [6]. Tumors that develop in the main pancreatic duct have a greater potential for malignant degeneration, whereas those located in the secondary pancreatic ducts, are more rarely described to undergo malignant transformation [8].

Histopathologically, IPMNs can be subclassified into four main subtypes: gastric, intestinal, pancreatobiliary and oncocytic. Among them, the gastric subtype most often affects the secondary ducts. The other subtypes are more likely to involve the main pancreatic duct, with the intestinal pattern subtype being the most frequent [9,18]. The pancreatobiliary IPMN subtype (IHC pattern: MUC1 and MUC5AC positive, MUC2 negative) has the worst prognosis [1]. The histopathological study of the patient in this report suggests that the MT-IPMN should be classified as an intestinal subtype (IHC pattern: MUC2 and MUC5AC positive, MUC1 negative). Pantano et al. analyzed the tumor specimens of 59 patients who underwent surgical resection for pancreatic ductal adenocarcinoma. The authors found that in univariate analysis, overall survival seems to be influenced by MUC2 expression [10]. The median survival time of patients with positive MUC2 expression was 44 months versus 16 months for patients with negative MUC2 expression, and the difference was statistically significant [10].

### 3.3 Diagnostic work-up

Since clinical findings are nonspecific, radiological assessment is central to the multidisciplinary management of a patient with IPMN [2,3]. Gadolinium-enhanced MRI with MRCP and endoscopic ultrasonography are recommended for better radiological characterization of the lesions and facilitates recognition of septae, duct communication and nodules. IPMNs are usually divided into three groups: MD-IPMN, when there is exclusive dilation of the main pancreatic duct above 5 mm, without an obstructive factor; BD-IPMN when the tumor originates from a branch of the main pancreatic duct, but there is communication with the main duct; and MT-IPMN, when the tumor compromises the main pancreatic duct and its branches [1,4,6].

The radiological report should include a targeted, systematic and comprehensive description of all anatomical site of abnormalities, such as “high-risk stigmata” (i.e., enhanced mural nodule 5 mm, main pancreatic duct size of 10 mm) and "worrisome features" (i.e., cyst of 3 cm, enhancing mural nodule <5 mm, thickened enhanced cyst walls, main pancreatic duct size of 5–9 mm, lymphadenopathy and a rapid rate of cyst growth > 5 mm/2 years) [1,2,4].

From a genetic point of view, mutations can be identified and may be related to the increased risk of malignant transformation for a given IPMN, including germline mutations in a pancreatic cancer susceptibility gene: *ATM*, *BRCA2*, *MSH6 and PALB2* [12]. Currently, several panels are available and can contribute to the decision-making process. Recent studies have also sought the relationship between somatic mutations and a greater possibility of malignancy by analyzing pancreatic juice samples collected through retrograde endoscopic cholangiopancreatography [14]. Schleger et al. have identified that deregulation of *c-MYC* protein may be involved in early neoplastic development and progression of pancreatic cancer, as well as a correlation between mutation rates and the histological grade of the neoplasia [15].

By associating the findings of histopathological studies with germline and somatic alterations, it will be possible to arrive at a more accurate diagnosis and to create a more tailored treatment based on the malignant potential of the lesion. In patients with a positive family history for IPMNs or pancreatic cancer, the analysis of germline mutations can be decisive in the management selection process, and these patients may warrant additional surveillance for lesions in the pancreatic remnant or other extrapancreatic tumors [[13], [14], [15], [16]].

### 3.4 Treatment modalities and prognosis

Cysts with obvious “high-risk stigmata”should undergo resection in surgically fit patients. All patients with cysts of 3 cm in size without “worrisome features” should undergo surveillance according to size stratification [[1], [2], [3]]. Among all subtypes, MT-IPMNs are the most challenging in terms of choosing the ideal therapeutic strategy. These lesions are the most difficult to treat because they are generally multifocal and compromise different locations of the pancreatic parenchyma. Choosing the appropriate surgery to treat MT-IPMN depends both on the distribution and characteristics of the lesions in the gland, as well as on the patient's clinical conditions and possible postoperative complications 3,4,6]. The patient in this report had an multifocal MT-IPMN with IPMNs located in the head, neck, body and tail of the pancreas. For this reason, we chose to perform histopathological studies of frozen sections during the surgical procedure to ensure that no main duct tissue had high-grade dysplasia or neoplasia where the anastomosis would be performed.

In patients who have undergone resection for IPMNs with negative margins, such as the patient in the present report, it is also important to survey for metachronous tumors. The development of invasive carcinoma in the remnant pancreas has been documented [1,7]. Miller et al. followed 153 patients who underwent resection of IPMNs with noninvasive pathology and without residual IPMN at the initial operation. During a mean follow-up of 73 months, 20% of these patients developed a new radiographic lesion consistent with IPMNs, and 10% of them were found to have invasive cancer [6].

In a multicenter Japanese study of 1074 IPMN patients, 14.4% developed postoperative recurrence at a median follow-up of 24 months, and the recurrence rate significantly increased as the grade of dysplasia increased. Preoperative symptoms, MD-IPMN type, IPMN located in the pancreatic body/tail, main pancreatic duct diameter (≥10 mm), mural nodules (≥5 mm) and high-grade dysplasia/invasive IPMN were found more often in patients who developed high-risk lesions in the pancreatic remnant. Moreover, surgery for metachronous high-risk lesions in the remnant pancreas seemed to improve survival [8].

The option to perform duodenopancreatectomy with observation of the residual pancreatic parenchyma was reinforced by the histopathological study of the specimen, which did not find the presence of pancreatic adenocarcinoma in the resected pancreatic segment. Currently, the patient is in good condition and has a productive life 4 years after surgery, and there has been no recurrence of the disease.

## 4 Conclusion

We consider that the MRI findings showing that the MD-IPMN portion was restricted to the head of the pancreas, normal Ca19.9 serum level, lack of a family history of pancreatic neoplasia, and small residual BD-IPMNs in the body/tail were indications for the surgical choice for the MT-IPMN presented in this case.

## Declaration of Competing Interest

The authors report no declarations of interest.

## Funding

None to report.

## Ethical approval

This study was performed in accordance with SCARE criteria and all the data and images were accessed in the hospital database, with the approval of the Ethics Committee (number: 33551020.8.0000.5514).

## Consent

We obtained written patient consent to publication.

## Author contribution

All authors contributed equally to this manuscript: study design, data collections and writing.

## Registration of research studies

Not necessary for this case report.

## Guarantor

Bassaneze T is the article guarantor.

## Provenance and peer review

Not commissioned, externally peer-reviewed.
